# Habitat monitoring and conservation prioritization of Western Hoolock Gibbon in upper Brahmaputra Valley, Assam, India

**DOI:** 10.1038/s41598-021-94844-8

**Published:** 2021-07-29

**Authors:** Kuladip Sarma, Malabika Kakati Saikia, Bidyut Sarania, Himolin Basumatary, Siddhartha Sankar Baruah, Bhrigu Prasad Saikia, Awadhesh Kumar, Prasanta Kumar Saikia

**Affiliations:** 1 Department of Zoology, Animal Ecology and Wildlife Biology Lab., Gauhati University, Gopinath Bordoloi Nagar, Jalukbari, Guwahati-14, Assam India; 2 Department of Zoology, Cotton University, Panbazar, Guwahati-01, Assam India; 3 Centre for Ecological Sciences, Theoretical Ecology and Evolution Lab., Indian Institute of Science, Bengaluru, Karnataka 560012 India; 4 Department of Environmental Science, Tezpur University, Napam, Tezpur, Assam 784028 India; 5 Department of Environmental Science, Gauhati University, Gopinath Bordoloi Nagar, Jalukbari, Guwahati-14, Assam India; 6 Department of Forestry, Northeastern Regional Institute of Science and Technology (NERIST), Nirjuli, Arunachal Pradesh 791109 India

**Keywords:** Biological techniques, Ecology, Zoology, Climate sciences, Ecology

## Abstract

The present study aimed at predicting the potential habitat of Western Hoolock Gibbon (*Hoolock hoolock*) in the upper Brahmaputra River Valley, Assam, India, and identifying priority conservation areas for the species, taking canopy cover into account. We used the maximum entropy algorithm for the prediction of the potential habitat of the gibbon using its current distribution with 19 environmental parameters as primary predictors. Spatio-temporal analyses of the habitat were carried out using satellite-based remote sensing and GIS techniques for two decades (1998–2018) along with Terra Modis Vegetation Continuous Field product to examine land use land cover (LULC), habitat fragmentation, Normalized Difference Vegetation Index (NDVI) and tree cover percentage of the study area. To identify the conservation priority area, we applied a cost-effective decision-making analysis using systematic conservation prioritization in R programming. The model predicted an area of 6025 km^2^ under high potential habitat, a major part of which was found to overlap with dense forest (80%), followed by moderately open forest (74%) and open forest (66%). The LULC change matrix showed a reduction of forest area in the predicted high potential habitat during the study period, while agricultural class showed an increasing trend. The fragmentation analysis indicated that the number of patches and patch density increased from 2008 to 2018 in the ‘very dense’ and ‘dense’ canopy regions of the gibbon habitat. Based on the conservation priority analysis, a 640 km^2^ area has been proposed to conserve a minimum of 10% of gibbon habitat. The current analysis revealed that in the upper Brahmaputra Valley most areas under dense forest and dense canopy have remained intact over the last two decades, at least within the high potential habitat zone of gibbons independent of the degree of area change in forest, agriculture and plantation.

## Introduction

Primate diversity and abundance in a forest site are affected by the structural variables or quality of the habitat and indirect/direct anthropogenic impacts^1–3^. Investigation of primate habitat and its insularisation is a major research concern in tropical forest ecosystem conservation^4^, as almost 90% of primate taxa, in general, are threatened by habitat fragmentation owing to their dependence on tropical forest^5,6^. The habitat requirements of a species are often considered to correlate with feeding strategy. Therefore, it is important to understand the ecological flexibility or limits of primate communities and species, to implement effective management strategies for their conservation^7–9^. In the Indian context, primates are rarely studied in terms of habitat requirements. Meanwhile, severe challenges to primate conservation such as habitat loss, fragmentation, and unsustainable economic development persist. Continuous monitoring of habitat and qualitative assessment of species’ preferred habitat parameters are urgent needs to improve prioritizing conservation action. In this paper, we present a case report on Western Hoolock Gibbon (*Hoolock hoolock*) in the upper Brahmaputra Valley with special reference to habitat monitoring.

Hoolock gibbons are found in several forest types of northeast India^10,11^, Bangladesh^10,12^, Myanmar^13^, and South China^14^. Although hoolock gibbons require habitats with an abundance of food, they also require forests with dense canopy^15^. The high rate of forest fragmentation and degradation poses a serious threat to the survival of Western Hoolock Gibbon (*Hoolock hoolock*), especially on the south bank of the Brahmaputra River in Assam^11,16^. Studies on gibbons in India have mainly concentrated on population and behavioural aspects, with very little emphasis on the quantification and monitoring of habitat parameters^17^. The frugivory of gibbons is well established^18–21^ with detailed information about food plants and food items throughout its distribution range. However, minimal efforts have been done to understand food plants’ association of hoolock gibbon in relation to forest structure^17^. Some research articles provide baseline ecological data of the species in northeast India^16,20,22^. As a number of primate species coexist in the region^23^, it is important to determine the specific habitat requirement of each species. Limited studies of sub-populations of gibbons in various pockets of northeast India’s forest cannot be extrapolated widely. Therefore, a landscape-level study on habitat with the application of advanced tools and techniques is the need of the hour. Currently, no studies have been reported on gibbon habitat monitoring using temporal satellite data from the region.

The present study aimed to investigate the site-specific preference of gibbon habitats in relation to canopy cover and forest cover by integrating a maximum entropy algorithm to predict potential habitat and geospatial tools to incorporate major habitat forms. The study also attempted to test the hypothesis that hoolock gibbons prefer dense forest and closed canopy. This information will provide quantified baseline data for the future conservation of gibbons in the upper Brahmaputra River stretch in Assam, India. Subsequently, we identified fragments of gibbon habitat that need to be prioritized for protection. We adopted integer linear programming techniques (prioritizr R package) for the decision-making analysis, considering the tree canopy as a cost for conservation^24^.

### Study area

**Figure 1:**
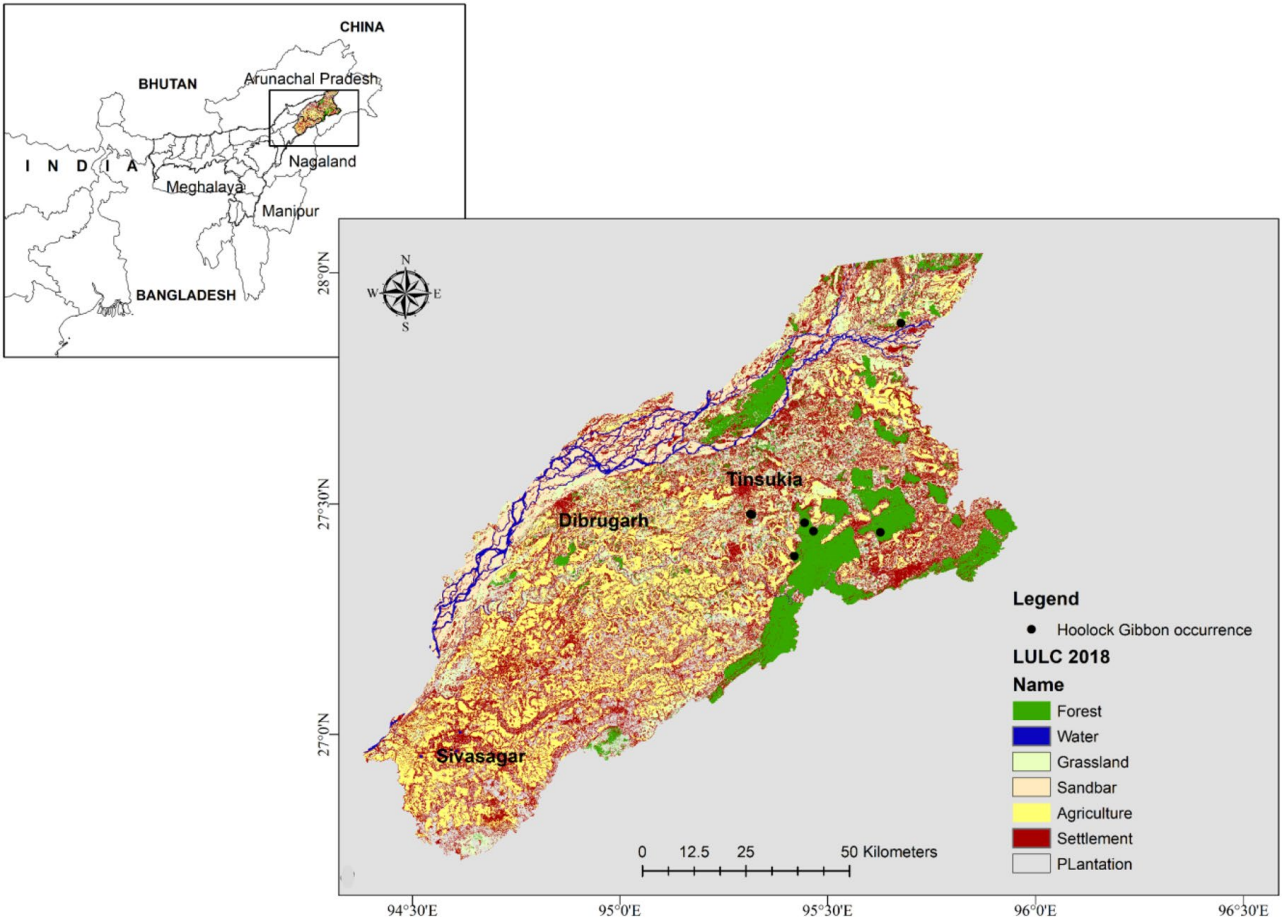
Land Cover map (2018) of the study area in the upper Brahmaputra Valley, Assam, India showing the locations of gibbon’s occurrence. Map prepared using ArcGIS 10.3.

The study was conducted in the upper Brahmaputra Valley, where the hoolock gibbon is distributed on the south bank of the Brahmaputra River. The southern part of the upper Brahmaputra Valley comprises mainly four districts of Assam: Jorhat, Sivasagar, Dibrugarh and Tinsukia. The study area lies between 94°30ʹ E to 96°0ʹ E longitude and 27°0ʹ N to 27°45ʹ N latitude, covering an area of about 9851 km^2^ (Fig. 1). Four major protected areas, including a national park, fall within this area, which signifies the importance of the landscape in terms of floral and faunal conservation priorities. The vegetation of the study area is identified as wet evergreen forest of the *Dipterocarpus*–*Mesua* series^25^. The climate of the Brahmaputra Valley as a whole is similar to the Southeast Asiatic Monsoon climate modified as per local physical conditions^26^. The valley receives as high as 3900 mm rainfall annually in the extreme northwest and northeast hilly tracts^27^. The mean annual temperature ranges from 23 to 24 °C.

## Methods

The survey was conducted from January 2017 to December 2018 for gibbon occurrence and ground verification (ground-truthing). Gibbon locations by both direct sightings and loud calls heard were collected with handheld GPS. Along with the occurrence locations, the land use pattern was also noted to prepare the LULC supervised classification signature. Occurrence data collected were used in the predictive model thereafter. The detailed flowchart of the methods used in the study is given in the supporting information (Appendix S1).

### Predictive distribution model

Maxent software version 3.1.0 (Computer Sciences Department– Princeton University, 2004) was used for predicting the potential distribution area of *H. hoolock.* Nineteen Environmental layers comprising annual mean temperature, temperature seasonality, isothermality, annual precipitation, precipitation seasonality, etc. (Supporting information; Appendix S2) and 23 species’ occurrence data were fed into Maxent, and the final predicted potential distribution area was projected using ArcGIS 10.3. The detailed explanation of Environmental Niche Modelling (ENM) using Maxent has been described by Phillips et al.^28^.

The occurrence data of *H. hoolock* was gathered through extensive field surveys conducted along the upper Brahmaputra Valley, Assam. A total of 23 independent distribution localities of gibbons were collected from the field, and all localities were used in the final modelling process. As Maxent employs maximum likelihood algorithm for presence only data of a species, there is no requirement of absence data for the same. The nineteen environmental layers were downloaded from the WorldClim website (www.worldclim.org) with spatial resolution of 30″ (seconds) (WorlClim version 2.1).

The Jackknife validation methodology was performed following the method developed by Pearson et al.^29^, which was shown to be effective for sample sizes of 25 or less. To avoid over-fitting of the test data, the regularization multiplier value was set at 0.1^30^. The maximum number of background points was 1000. Linear quadratic and hinge features were used. We selected 80% of the data for training and the remaining 20% for testing, and 10 percentile threshold rules was employed. A total of 100 runs were set for model building^31^. AUC (**A**rea **U**nder the receiving operator **C**urve) was used to test the model’s goodness-of-fit, with the highest AUC value considered as the best performer. The contributions of the variables were assessed through the Jackknife procedure. The final output was divided into three potential distribution areas that were regrouped based on the natural breaks available in reclassification tool of Arc Map 10.3 with a range of 0–1: Low potential (< 0.32); Moderate potential (0.32–0.47); High potential (> 0.47).

### Preparation of land use land cover (LULC) maps and change detection analysis

#### Data acquisition

Landsat satellite imageries were downloaded from the USGS Earth Explorer viewer (US Geological Survey, https://earthexplorer.usgs.gov) to determine the LULC classes and analyse their change over a span of 20 years. The satellite data were of variable dates, selected based on the quality of the data, data availability, and dry season. A total of six Landsat imageries of the same spatial resolution (30 m) were acquired for the years 1998 (Landsat 5), 2008 (Landsat 5) and 2018 (Landsat 8) (Supporting information; Appendix S3). The software packages used in the analyses were ERDAS Imagine 2011, ArcGIS 10.3, and QGIS 3.14.

#### LULC classification and change detection

A hybrid classification technique was adopted to digitally categorize each Landsat image, since this technique has been shown to perform better in the case of spectral variability of individual land cover features^32–34^. Several studies have suggested that hybrid classification produces superior results compared to unsupervised or supervised classification alone^35,36^. The downloaded satellite images were assigned per-pixel signatures and based on the specific DN value of each landscape element, seven different LULC classes were decided, viz. agriculture, grassland, forest, plantation, sandbar, settlement, and water (Supporting information; Appendix S4).

However, scrubs have been included in the forest class and not been classified separately, since, scrubs are located in periphery of forests and are in continual transition with the open forest class. Further, the scrublands are small in size (area), and during filtering we had to merge those patches with the nearest land cover class. A vector polygon was drawn around each representative predetermined LULC class (based on ground-truthing) to prepare the spectral signatures with minimal confusion points^37^. A total of 70 spectral signatures for the respective LULC classes derived from the satellite imagery were recorded using the pixels enclosed by these polygons. These spectral signatures were then used to reclassify the images using a maximum likelihood classification algorithm that classifies the pixels based on the maximum probability of belonging to a particular class^38^. Then, the classified images were filtered using a neighbourhood majority function.

The LULC change-transition matrix was computed using the overlay procedure in ArcGIS to quantify the area converted from one particular LULC class to another during the study period. Later, visual interpretation was used to address the mixed pixels problem^34^.

#### Accuracy assessment

The non-parametric Kappa test was performed to measure classification accuracy to account for diagonal elements and elements in the confusion matrix^39^. A confusion matrix was constructed with each row representing LULC classes in the classified map and columns representing the reference LULC classes. The kappa co-efficient was determined from this matrix for each classified map. Kappa Co-efficient is the degree of agreement or precision between the classified map and the reference data^39,40^.

### Normalized difference vegetation index (NDVI)

NDVI was calculated using the infrared band (0.77–0.86 mm) and red band (0.62–0.68 mm) of the Landsat image of the year 2018 to compare the forest cover of the classified image. The NDVI images have been reclassified into three forest cover categories: Open forest, Moderately Open forest and Dense forest, based on natural breaks given by the reclassification tool of ArcMap 10.3.

### Percentage canopy cover

Terra Modis Vegetation Continuous Field (VCF) product was used to extract the percentage tree cover of the study area. VCF products is a monthly composite of Terra MODIS 250 m and 500 m Land Surface Reflectance data, and the products provide a gradation of percent tree cover (percent of a pixel covered by canopy), percent non-tree vegetation (non-tree canopy pixel), and percent non-vegetated (pixel with no vegetation) (https://lpdaac.usgs.gov). We constructed a composite image of tree canopy cover (mean aggregated) for the years 2008 and 2018 on the Google Earth Engine platform (GEE). GEE is a platform that allows users to compute its multi-petabyte catalogue of satellite imagery and geospatial dataset at planetary-scale, and export the analysis in Geotiff format or tabulated data^41^. We exported the percentage tree cover (250-m spatial resolution) as a TIFF image and reclassified it into three categories following Forest Survey of India (FSI) forest cover classification (www.fsi.nic.in), viz. no canopy (0–10%), open canopy (10–40%), dense canopy (40–70%), and very dense canopy (> 70%). We used ArcGIS for the reclassification and extraction of pixel data.

### Patch analysis and conservation prioritization

FRAGSTATS v3.3^42^, a spatial pattern analysis tool, has been used to quantify class or patch metrics from the canopy cover raster of 2008 and 2018 cropped for high potential gibbon habitat. The patch or fragments statistics given by FRAGSTATS, viz. patch density (PD), mean patch size (MPS), percentage of landscape (PLAND), etc., are very informative indices for quantitative comparison of fragments within the landscape.

To minimize over-prediction of the Maxent model in terms of conservation planning, the prioritizr ^43^ package in the R platform was used to find out the potential feasible areas for conservation action within the high-potential zone of the predicted distribution map. Here conservation ‘problem’ is defined as any ecological or logistical shortcomings/obstacles that could detract from the larger purpose of conservation. When describing a conservation problem, it's important to include both the economic and ecological costs of conservation. The conservation cost refers to the expense of restoring the species' habitat for it to survive in the future. For example, if a given area's canopy percentage is very low, it might be wiser to choose another adjacent location with a larger canopy percentage to reduce the conservation authority's managerial costs. Furthermore, in addition to other socio-political factors (e.g. land acquisition), the time and monetary investment required to restore a low canopy region to a higher canopy area will be greater. In the prioritizr R package, we define conservation ‘problem’ based on two variables, namely, canopy percentage and percentage of human settlement in the gibbon’s habitat. Considering the total area, a 4-km × 4-km grid map was overlaid on the high potential distribution zone, and grids within the protected areas were excluded using the ‘locked out’ function in the conservation problem definition. Also, grids with more than 30% human settlement areas were excluded for feasible conservation planning of gibbon protection in the region. The cost of conservation was assessed based on percentage canopy present in each grid in a scale of 1 to 5 (5 = 0–20%; 4 = 20–40%; 3 = 40–60%; 2 = 60–80%; 1 = >80%). The lowest canopy percentage grid was assigned a scale of 5 and the highest was assigned 1. We assigned three different threshold percentages accounting for 10%, 15% and 20% of the species distribution area that was intended to be conserved, and the ‘problem’ was defined accordingly. The output maps were then checked for irreplaceability and final maps were prepared.

## Results

### Predictive distribution of Western Hoolock Gibbon

**Figure 2:**
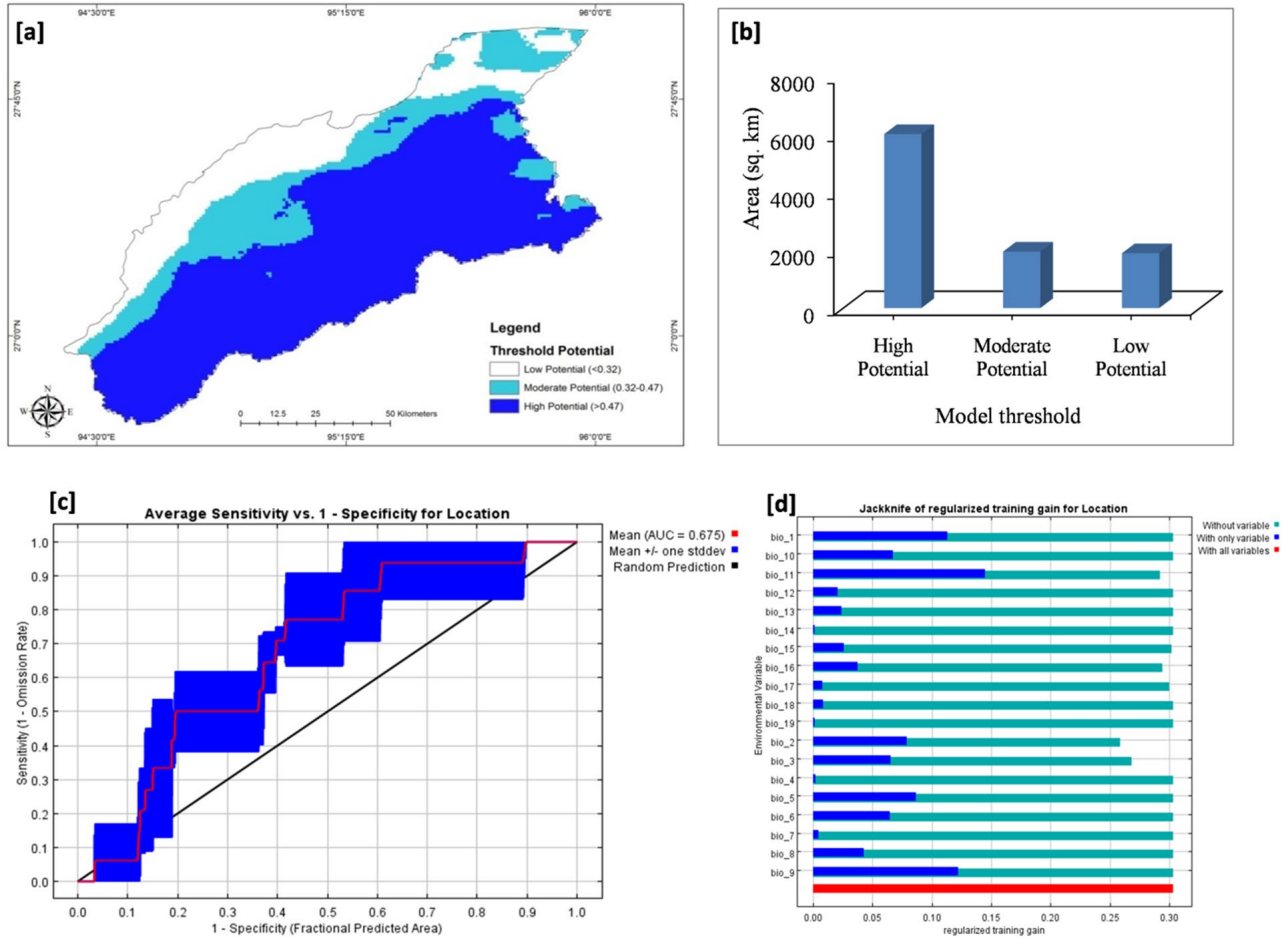
Maxent results (**a**) predictive distribution map prepared using Maxent 3.1 and ArcGIS 10.3 (**b**) area statistics in different model thresholds (**c**) ROC curve and (**d**) Jackknife validation output.

The predictive distribution model (Maxent) has shown average model performance with lower AUC value (AUC = 0.675) (Fig. 2a, c, d). The presence-only model depicted the highest importance of mean temperature in the coldest quarter of the year (Bio11; 30.7%) out of the all 19 different bioclimatic variables used in the model (Supporting information; Appendix S1). This may be due to food availability as the coldest quarter of the year represents the lean period of food availability. So, depending on the fall back food available in the habitat, the distribution of the species varies.

The output predictive map has shown that the high potential habitat zone comprised the highest area (6025 km^2^) followed by moderate (2003 km^2^) and low potential (1729 km^2^) habitat zones (Fig. 2b).

### LULC change matrix

Accuracy assessment showed that the kappa values of the LULC classifications were 0.764, 0.693, and 0.757 for 1998, 2008, and 2018 respectively. LULC analyses revealed that agriculture, grassland and settlement classes covered majority of the study area (9851 km^2^; Table 1; Appendix S5). Plantation and settlement showed increase in percentage area between 1998 and 2018, indicating a significant role of humans in land conversion. Urbanization and population expansion are major reasons for the continual surge in settlement areas (102%) (Table 1). On the contrary, forest area showed a slight decline (4.5%) during the two decades. Area decrease in forests is expected to be higher, but due to the inclusion of scrubland in the forest class, the resultant area decline might have been biased lower. It is important to note here that the matrix table (Supporting information; Appendices S6 and S7) shows the area conversion from one year to another year (e.g. 1998–2008 and 2008–2018). An area of a class can either gain/lose area from/to another class. It is an inter-conversion process. For instance, grassland can lose an area (convert) to forest through secondary vegetation succession; at the same time, it can also gain area from waterbodies (through shrinking water) or sandbar (through primary vegetation succession).

**Table 1:** Area information and change of the LULC classes in the study area (sq. km) during 1988, 2008 and 2018.

LULC class	Year			Percentage change (%)		
	1998	2008	2018	1998–2008	2008–2018	1998–2018
Agriculture	2379	2633.9	1885.4	10.7	− 28.4	− 20.7
Forest	1157.2	1063.9	1105.2	− 8.1	3.9	− 4.5
Grassland	2667.4	1744.6	1894.3	− 34.6	8.6	− 29.0
Plantation	934.3	1367.6	1142.5	46.4	− 16.5	22.3
Sandbar	1001.5	699.5	675.7	− 30.2	− 3.4	− 32.5
Settlement	1443.5	2019.6	2915.5	39.9	44.4	102.0
Water	268.3	322.1	232.6	20.1	− 27.8	− 13.3

*Negative (−) sign indicates percentage decrease.

All the classes showed inter-conversion among classes in both study periods: 1998–2008 and 2008–2018 (Supporting information; Appendices S6 and S7). The maximum addition of area from other classes occurred in the settlement, agriculture, grassland, and plantation in both periods. Conversion of areas from forest to anthropogenic utilization classes (agriculture, plantation, and settlement) accounted for 300.5 km^2^ and 177.1 km^2^ during 1998–2008 and 2008–2018 respectively (Supporting information; Appendices S6 and S7). On the other hand, forest area increased by 261.5 km^2^ and 266.4 km^2^ from other LULC classes in 1998–2008 and 2008–2018 respectively.

**Figure 3:**
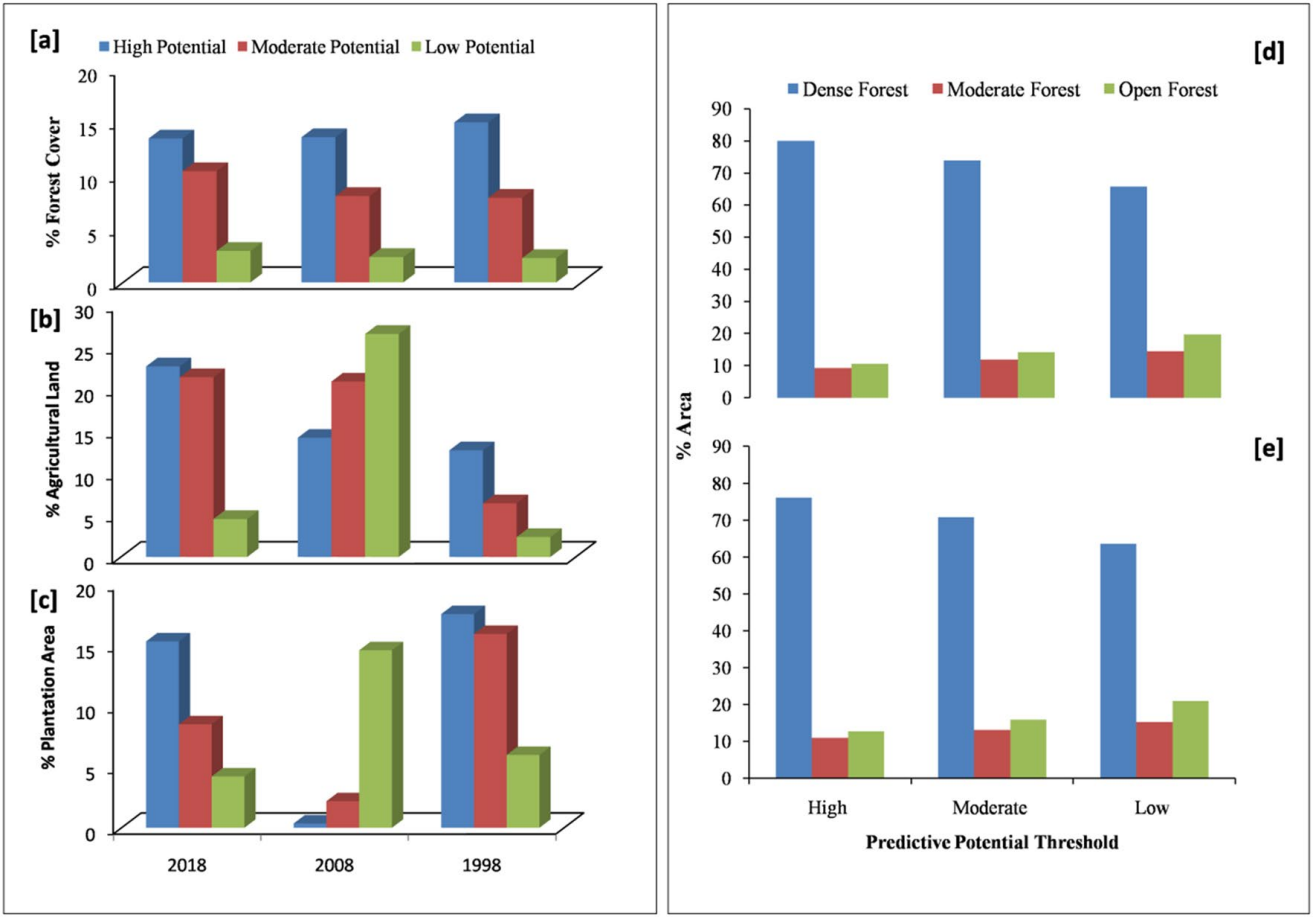
Percentage area of (**a**) forest (**b**) agriculture and (**c**) plantation within different predictive thresholds in the year 2018, 2008, and 1998 and percentage area of dense forest, moderate forest, and open forest derived from NDVI within different predictive thresholds (**d**) including tea plantation (**e**) omitting tea plantation.

The distribution of forest cover among these thresholds was analysed and it was observed that the highest percentage of forest cover was within the high potential habitat zone in the year 1998, which gradually decreased in 2008 and 2018 (Fig. 3a). Another important land use class, i.e., agriculture was highest in 2018 within the high potential habitat zone (22.71%) of gibbons, which showed an increasing trend from 1998 onwards (Fig. 3b). A similar trend was observed in moderate potential zone (6.41%, 20.89%, 21.41% for the years 1998, 2008 and 2018 respectively). However, in the low-potential habitat zone, agriculture covered the highest percentage area in the year 2008 (26.58%). Plantation area displayed a minimal decrease in area from 1998 to 2018 in all the three potential thresholds (Fig. 3c).

### NDVI and potential Gibbon distribution

The NDVI map of 2018 (Supporting information; Appendix S8) shows that the maximum tea plantation area (1148 km^2^) has been included in the total dense forest area (4312 km^2^) that is classified from NDVI. Dense forests and tea plantations have similar reflectance values and therefore, NDVI map shows tea plantations as dense forests. Lesser areas of tea plantation were included in moderate (58.92%) and open forest (60.65%) (Supporting information; Appendix S9).

Overall areas of NDVI classes under various potential habitat zones of gibbons in the study area revealed that 80.04% of dense forest was under high potential habitat (Fig. 3d). In the moderate potential threshold, 73.90% of dense forest was included, and 65.82% of dense forest was found in the low potential threshold. While comparing NDVI classes (omitting the areas of tea plantation in predictive distribution thresholds), it was found that 76.21% of dense forest area was under a high potential threshold (Fig. 3e). In the moderate potential threshold, 70.83% dense forest was recorded, and 63.69% dense forest was found the in low potential threshold. Thus, the dense forest with tea plantation had a higher percentage in the high potential habitat zone (Supporting information; Appendix S10).

### Tree canopy cover change matrix

The canopy cover change matrix has shown that 48.8 km^2^ and 2.4 km^2^ area of the very dense canopy were converted to the dense canopy and open canopy respectively during 2008–2018 (Supporting information; Appendix S11). At the same time, 205.2 km^2^ of dense canopy was converted to an open canopy and 368.6 km^2^ of the open canopy to no canopy. Chronological conversion from the very dense canopy to dense canopy, dense canopy to open canopy, and open canopy to no canopy is the result of decrease in tree density and canopy senescence. Conversely, an increase in canopy cover was also observed, probably due to canopy growth, which showed area conversion from no canopy to open canopy (724.3 km^2^), open canopy to dense canopy (828.3 km^2^) and dense canopy to very dense canopy (199.3 km^2^).

While comparing tree canopy in the predictive potential habitat of gibbons during the period of 2008–2018, we found that very dense canopy increased by 141.3 km^2^ in the high potential habitat zone, whereas the open canopy decreased by 41.3 km^2^ (Table 2). The highest increase in canopy area was evident in the dense canopy in the moderate potential habitat zone (439.3 km^2^).

**Table 2:** Canopy cover in different potential habitat in 2008 and 2018 (area in sq. km).

Canopy cover	2018			2008			2018–2008		
	HPH	MPH	LPH	HPH	MPH	LPH	HPH	MPH	LPH
NC	52.7	106.9	1113.9	591.8	146.8	890.9	− 539.1	− 39.9	223.0
OC	4042.6	1509	657.2	4083.9	1504.5	891.9	− 41.3	4.5	− 234.7
DC	1219.8	267.8	91.5	780.5	234.2	91.5	439.3	33.6	0.0
VDC	694.1	65.6	30.1	552.8	63.2	19.2	141.3	2.4	10.9

No canopy (NC), Open canopy (OC), Dense canopy (DC), Very dense canopy (VDC), High potential habitat (HPH); Moderate potential habitat (MPH) and Low potential habitat (LPH).

### Protected area coverage

Overall, the protected area covers 20.82% of the study area (Supporting information; Appendix S12), which includes 17.73% of the total high potential gibbon habitat, 27.51% of moderate potential habitat and 23.74% of the low potential habitat. Out of the total, 95.03% of very dense canopy in the study area was within the protected range in 2018 whereas the same was 97.19% for the year 2008. But when compared to the percentage of the total area irrespective of canopy categories, only 7.62% of the area was under very dense canopy within protected areas in 2018. Likewise, for the year 2008, the percentage of the same was 6.27% (Supporting information; Appendix S13). Furthermore, within the protected range, 75.35 km^2^ has been lost from dense canopy area from 2008 to 2018 whereas the very dense canopy increased by 133.17 km^2^ during the same decade. In contrast, in the non-protected range, a significant increase in dense canopy forest was recorded (548.25 km^2^) from 2008 to 2018. Very dense canopy was also found to increase in the non-protected range (21.43 km^2^), but the area addition was comparatively lower than that in the corresponding protected range.

### Patch analysis and conservation recommendations

The FRAGSTATS result showed an increase in the number of fragments in both dense and very dense canopy forests from 2008 to 2018 within the high potential gibbon distribution zone (Supporting information; Appendix S14). In 2008, the mean patch sizes in dense and very dense canopy areas were 1.18 km^2^ and 8.02 km^2^ respectively. However, the same was observed to be marginally higher in 2018. The contiguity index of patches of very dense canopy was 0.36 in 2018, and slightly decreased in the year 2018 to 0.34. The mean Euclidian Nearest Neighbour Distance among patches was found highest within very dense canopy forest (1646.21 m) in the year 2018.

**Figure 4:**
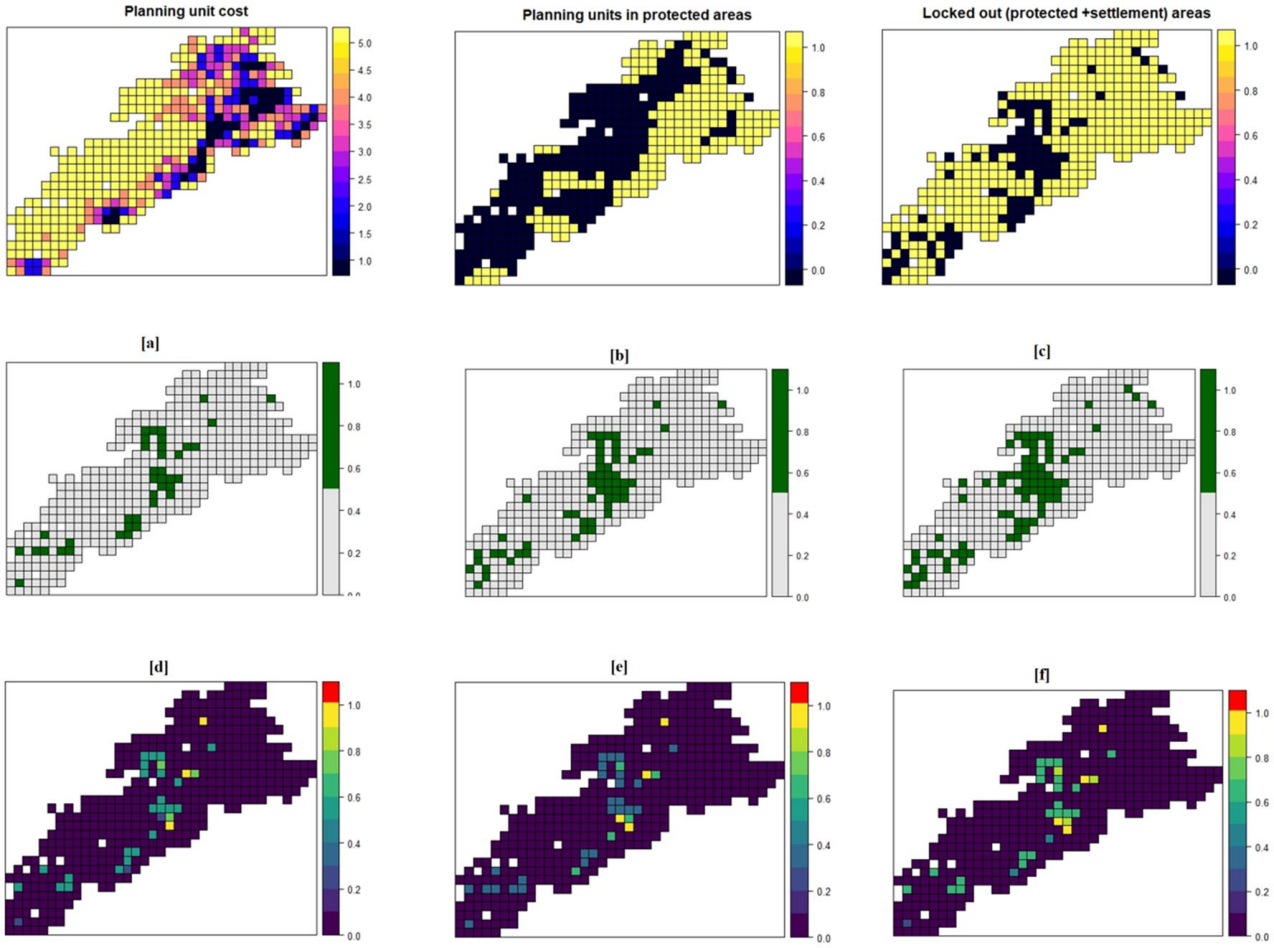
Proposed conservation planning units (4 km × 4 km grids) in the high potential gibbon habitat of upper Brahmaputra Valley, Assam, India (prepared in prioritizr R package, Rstudio platform^43^). All protected areas are excluded from the grid map as those are already under prioritized category. Settlement percentage was calculated for each grid cell and the grids having more than 30% settlement land use were excluded from the analysis: (**a**) to protect 10% of gibbons’ habitat; (**b**) to protect 15% of gibbons’ habitat; (**c**) to protect 20% of gibbons’ habitat; (**d**); (**e**) and (**f**) shows irreplaceability of the proposed units to conserve 10%, 15% and 20% gibbons’ habitat respectively (higher the value, more is the irreplaceability; planning replaceable units are shown in purple, blue, green, and yellow colours, and truly irreplaceable planning units are shown in red colour).

The proposed conservation plan to conserve 10% of high potential habitat, excluding both existing protected areas and high human habitation areas (> 30%), showed 40 grids (640 km^2^) highlighted for conservation (Fig. 4a–f). Similarly, 58 grids (928 km^2^) and 81 grids (1296 km^2^) were highlighted to protect 15% and 20% of the species’ habitat, respectively.

## Discussion

Gibbon population and habitat monitoring are crucial owing to their declining populations. The present study has revealed the potential impact of forest dynamics in gibbon distribution and survival in the upper Brahmaputra Valley located along the south bank of the Brahmaputra River in Assam. The study area is one of the major habitat zones of western hoolock gibbon in India. Despite most of the forested areas are being legally protected, the heterogeneity of the forest composition and anthropogenic intrusion still remain a major concern. The LULC analyses revealed that agriculture, grassland, and settlement classes covered most of the study area. Plantation and settlement classes showed an increase in percentage areas between 1998 and 2018, suggesting a substantial anthropogenic role in land conversion. Urbanization and population expansion are major reasons for the continual surge in settlement areas. In contrast, forest area showed a slight decline (4.5%) during the two decades, the decrease in forest area was expected to be higher, but due to the inclusion of scrublands in the forest class in the current study, the resultant area decline might have appeared lower. It is also reported that fragmentation is severe in the lowland rainforest of the upper Brahmaputra Valley, and one-third of forest cover area has been lost in the last century^23^. The high percentage change in forest cover between 1998 and 2008 reflects the ineffective efforts of habitat conservation in the study area. As gibbons are known to prefer dense or pristine forests for living, a major concern for conservationists is to protect dense forest areas. The shrinkage of forest cover in fragments of known gibbon habitat in the study area has also been reported in earlier studies^16,23,44^.

In the present study, the predictive potential habitat of the species is used as a primary tool to compare the habitat of the species on a temporal scale with particular reference to the forest area and canopy cover. Remote sensing methods used in studying the landscape and other socio-political disturbances in the study area have also proved to be effective in tracking biodiversity^45,46^. Nevertheless, ground verification is essential for better confidence in the results. Population data on western hoolock gibbons in the study area are readily available, as the upper Brahmaputra Valley is considered to be a major hotspot of gibbon distribution in India^16,20,22,47^. However, the study area lacks information on the major habitat parameters of the species.

The Maxent model predicted that the area under the high potential habitat zone in the study area was as high as expected. However, the Maxent model does not account for the historical distribution of species nor does it recognize physical barriers restricting the range of the species^48,49^. This may be the explanation for the inclusion of new areas of the species' high potential habitat dependent on bioclimatic suitability. The same argument was also supported by Sarma et al.^50^ in the predictive distribution of the eastern hoolock gibbon in Arunachal Pradesh, India. Moreover, extirpation of gibbons and other coexisting primates from several small fragments of the upper Brahmaputra Valley^16,23,44,51^ demand further study of localized specific habitat needs of the primates in general, particularly mating and dispersal requirements.

The decreasing trend of forest cover within the high potential habitat of gibbons in the study region over the last two decades and the higher percentage of agriculture in the same habitat zone highlights the intrusive presence of agricultural matrix in gibbon’s habitat. Sarma et al.^52^ also recorded the presence of agricultural land within the potential habitat of the eastern hoolock gibbon in Arunachal Pradesh. As the intrusion of agricultural lands creates fragments, it becomes more difficult for the species to explore all the resources available in different forest patches. Therefore, in the long run, the population health is likely to deteriorate.

Nonetheless, the two species of gibbons found in northeast India are reported to occur in forests of variable gradients in terms of quality and structure, and the present study is consistent with several such studies^16,17,20,21,23,44,47^. The present study also confirmed that the highest percentage area of dense forest was found in the high potential habitat zone of gibbons. We note that tea plantation is a key driver that affects the NDVI map of the study area, as tea plantations are classified under dense forest categories. Since we have omitted the tea plantation area from the dense forest category by testing the output map with ground data, the high potential habitat zone still represents the maximum dense forest area even if the tea plantation area is removed.

The canopy cover change of the study area in the last decade (2008–2018) carries a pessimistic outlook due to the high change in the very dense canopy. Chronological conversion from very dense canopy to dense canopy, dense canopy to open canopy, and open canopy to no canopy might be the result of canopy senescence, and decrease in tree density driven by increasing settlements. Conversely, an increase in canopy cover was also observed, probably due to canopy growth and vegetation succession, which showed area conversion from no canopy to open canopy (724.3 km^2^), open canopy to dense canopy (828.3 km^2^), and dense canopy to very dense forest (199.3 km^2^). While the present study shows minimal conversion of very dense canopy to open canopy (2.4 km^2^), it is of serious concern to the survival of gibbons given the habitat suitability of the study area. Although the increase in the area of very dense canopy (141.3 km^2^) within the high potential zone of gibbons over the last decade is encouraging, it is equally important to investigate the precise locations where the canopy has improved. Canopy contiguity is stated to be low in non-protected fragments of the upper Assam valley^16^. Interestingly, more than 80% of the high-potential gibbon habitats do not have legal protection, and there is a hopeful sign of an increasing trend in dense and very dense canopy in non-protected regions in the study area. However, the results indicate that protected areas of the upper Brahmaputra Valley, especially those within the high-potential zone of gibbon habitat, have retained the very dense canopy category with minimal change over the last decade to ensure gibbon survival. It may also be argued that gibbons are still more common in dense forests, while there is evidence of gibbons occurring in some rural inhabited areas of the upper Brahmaputra Valley. However, in northeast India, more than 50 percent of gibbon habitat is legally protected^15^ while the present study reveals that only 17.73% of the high potential habitat is under legal protection. Out of the 96 reserve forests  in Assam where gibbons have been reported^53^, the present study area includes as many as 42. This study, therefore, strongly supports the implementation of stringent laws on small fragments of rainforest in the upper Brahmaputra Valley for the potential survival of gibbons, taking into consideration that the species prefers dense forests. Gibbons are still present in small isolated fragments of the study area, irrespective of the canopy structure or the condition of the habitat. Because of the remnant geographical range of western hoolock gibbon, the species must survive with reduced resources before ecosystem conditions are adequately improved.

Our analysis shows that over the last two decades in the upper Brahmaputra Valley, Assam, most areas of dense forest, as well as the dense canopy, have remained intact, at least within the high-potential habitat of gibbons independent of the degree of change in forest, agriculture and plantation cover. The protected area network within the study area is of greater value in ensuring improved protection of the remaining gibbon habitat in the study area. However, other possible factors, such as food preference and availability, may be critical for defining the species' preferred habitat. It is noteworthy that quality of the forest is not directly related to forest density. For example, dense forest in higher altitude may lack sufficient succulent fruit trees for the gibbon diet. The decadal changes in forest cover and canopy structure of the study area therefore may be a few of the many drivers that affect the habitat preference of gibbons. On the other hand, knowledge of the population dynamics of the species in the study area can also lead to study of the unique features of the species' ecology, as the species is included in the Endangered category of the IUCN Red List. Here, in this study, we did not consider the physical conditions of the habitat and the constant land-use pressure posed by the human population on the habitat of the species. Though proposing future conservation areas within the predicted high potential zone of gibbons, we deliberately omitted the current protected areas, believing that the species within protected areas are effectively protected. The human settlement areas were filtered to ensure ease of implementing the Conservation Action Plan. However, the cost of conservation was objectively identified based on the fact that gibbons prefer dense canopy for survival. We also believe that this study provides the basis for a comprehensive investigation of factors influencing the responses of gibbons to environmental and climatic changes across their distributional range.

## Supplementary Information

Supplementary Information.
