# Supplementary material for: Habitat monitoring and conservation prioritization of Western Hoolock Gibbon in upper Brahmaputra Valley, Assam, India
Source: Sci Rep. 2021 Jul 29;11:15427. doi: 10.1038/s41598-021-94844-8 (PMC8322352; doi:10.1038/s41598-021-94844-8)
Supplement: Supplementary file 1 — Supplementary Information. [file 41598_2021_94844_MOESM1_ESM.docx]

**APPENDICES**

Appendix S1: Flow chart of methods used in the study.

Appendix S2. Environmental and its associated variables used in Maxent modelling and their percentage contribution in the model.

| **Code** | **Environmental variables** | **Unit** | **Percentage**  **Contribution** | **Percentage**  **permutation** |
| --- | --- | --- | --- | --- |
| Bio1 | Annual mean temperature | ◦C | 9.7 | 0 |
| Bio2 | Mean diurnal range (mean of monthly max. and min. temp.) | ◦C | 13 | 38.7 |
| Bio3 | Isothermality ((Bio2/Bio7) × 100)* |  | 19 | 16.2 |
| Bio4 | Temperature seasonality (standard deviation ×100) |  | 0 | 0 |
| Bio5 | Maximum temperature of warmest month* | ◦C | 6.8 | 0 |
| Bio6 | Minimum temperature of coldest month | ◦C | 0 | 0 |
| Bio7 | Temperature annual range (Bio5–Bio6) | ◦C | 0 | 0 |
| Bio8 | Mean temperature of wettest quarter | ◦C | 1.3 | 0 |
| Bio9 | Mean temperature of driest quarter* | ◦C | 0 | 0 |
| Bio10 | Mean temperature of warmest quarter | ◦C | 2.3 | 0 |
| Bio11 | Mean temperature of coldest quarter | ◦C | 30.7 | 14.1 |
| Bio12 | Annual precipitation | mm | 0 | 0 |
| Bio13 | Precipitation of wettest period | mm | 1.2 | 0 |
| Bio14 | Precipitation of driest period* | mm | 0 | 0 |
| Bio15 | Precipitation seasonality* | mm | 7.7 | 4.9 |
| Bio16 | Precipitation of wettest quarter | mm | 5.4 | 13.8 |
| Bio17 | Precipitation of driest quarter | mm | 2.4 | 11.9 |
| Bio18 | Precipitation of warmest quarter | mm | 0 | 0 |
| Bio19 | Precipitation of coldest quarter* | mm | 0.5 | 0.5 |

Appendix S3. Details of data used in the study

| **Year** | **1998** | | **2008** | | **2018** | |
| --- | --- | --- | --- | --- | --- | --- |
| **Satellite Data** | Landsat 5 TM | | Landsat 5 TM | | Landsat 8 OLI | |
| **Path/Row** | 134/41 | 135/41 | 134/41 | 135/41 | 134/41 | 135/41 |
| **Resolution** | 30m | 30m | 30m | 30m | 30m | 30m |
| **Date** | 26/12/1998 | 17/12/1998 | 21/02/2008 | 28/02/2008 | 16/02/2018 | 22/01/2018 |
| **Bands Used** | 5, 4, 3 | 5, 4, 3 | 5, 4, 3 | 5, 4, 3 | 5, 4, 3 | 5, 4, 3 |
| **Atmospheric correction** | Done | Done | Done | Done | Done | Done |

Appendix S4. Land use land cover classification scheme used in the study

| **Sl/No.** | **Class** | **Description** |
| --- | --- | --- |
| 1. | Forest | Areas under dense, open forest and scrublands |
| 2. | Settlement | Areas under urban and rural built-up including homestead area |
| 3. | Sandbar | Sandbars and other sand deposition areas including both dry and wet sand areas |
| 4. | Agriculture | Areas under cultivation including current fallow areas |
| 5. | Grassland | Areas dominated by grasses including vegetated sandbars and grazing areas |
| 6. | Plantation | Forest and agricultural plantations like orchards and tea gardens with significant patterns |
| 7. | Water | Water features such as river, stream, lakes and reservoirs. |


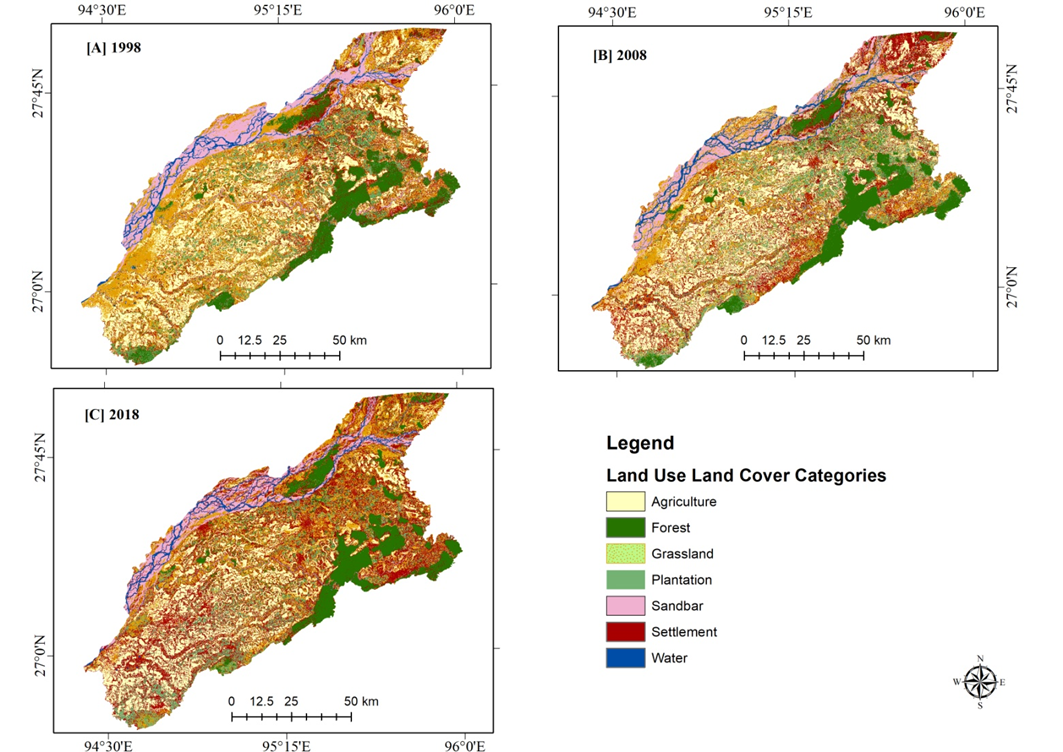


Appendix S5. Change detection maps of the study area [a] 1998, [b] 2008 & [c] 2018 (prepared in ArcGIS 10.3).

Appendix S6. Inter-conversion of Land Use Land Cover (LULC) classes between 1998 and 2008 (Area in km^2^)

| **1998** | LULC classes | **2008** | | | | | | | |
| --- | --- | --- | --- | --- | --- | --- | --- | --- | --- |
|  |  | Agriculture | Forest | Grassland | Plantation | Sand  bar | Settlement | Water | Grand Total |
|  | Agriculture | 1794 | 1.6 | 242.7 | 46.9 | 29.1 | 262.1 | 2.6 | 2379 |
|  | Forest | 18.3 | 802.4 | 47.6 | 169.1 | 3.2 | 113.1 | 3.5 | 1157.2 |
|  | Grassland | 550.1 | 27.7 | 756.6 | 417.4 | 78.9 | 802.3 | 34.4 | 2667.4 |
|  | Plantation | 57.2 | 88 | 175.8 | 427 | 13 | 172.1 | 1.2 | 934.3 |
|  | Sandbar | 47 | 1.2 | 252.8 | 22.7 | 424.2 | 60 | 193.6 | 1001.5 |
|  | Settlement | 164.6 | 141.3 | 220.5 | 282.3 | 22 | 601.9 | 10.9 | 1443.5 |
|  | Water | 2.7 | 1.7 | 48.6 | 2.2 | 129.1 | 8.1 | 75.9 | 268.3 |
|  | Grand Total | 2633.9 | 1063.9 | 1744.6 | 1367.6 | 699.5 | 2019.6 | 322.1 | 9851.2 |

Appendix S7. Inter-conversion of Land Use Land Cover (LULC) classes between 2008 and 2018 (Area in km^2^)

| **2008** | LULC classes | **2018** | | | | | | | |
| --- | --- | --- | --- | --- | --- | --- | --- | --- | --- |
|  |  | Agriculture | Forest | Grassland | Plantation | Sandbar | Settlement | Water | Grand Total |
|  | Agriculture | 1498.3 | 7.9 | 545.7 | 162.4 | 16.7 | 396.5 | 6.4 | 2633.9 |
|  | Forest | 1.3 | 838.8 | 44.9 | 67.5 | 1.8 | 108.3 | 1.3 | 1063.9 |
|  | Grassland | 175 | 32.7 | 509.9 | 262.4 | 138.4 | 574.5 | 51.7 | 1744.6 |
|  | Plantation | 20.7 | 107.5 | 249.2 | 414.4 | 8.6 | 563.1 | 4.1 | 1367.6 |
|  | Sandbar | 26.1 | 3.3 | 151.7 | 11.6 | 307.6 | 116.3 | 82.9 | 699.5 |
|  | Settlement | 163.7 | 112.8 | 373.4 | 223.8 | 25.9 | 1108.4 | 11.6 | 2019.6 |
|  | Water | 0.3 | 2.2 | 19.5 | 0.4 | 176.7 | 48.4 | 74.6 | 322.1 |
|  | Grand Total | 1885.4 | 1105.2 | 1894.3 | 1142.5 | 675.7 | 2915.5 | 232.6 | 9851.2 |

**
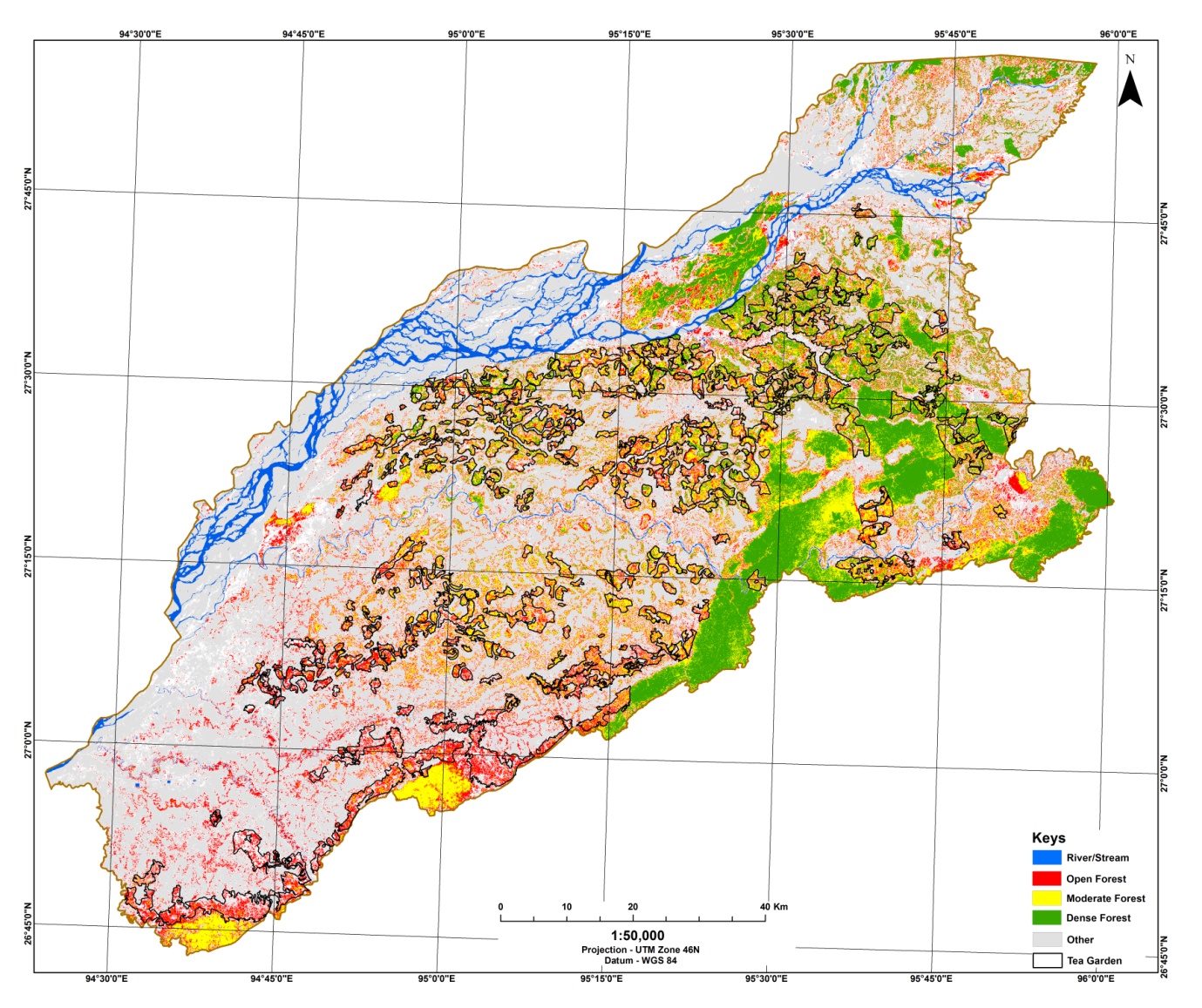
**

Appendix S8. NDVI map of the study area calculated from the Landsat image of 2018 (prepared in ArcGIS 10.3).

Appendix S9. Overall area (in km^2^) vs. tea plantation area of NDVI classes in the study area.

Appendix S10. Percentage area of NDVI classes in high potential model threshold in the study area with and without tea plantation

Appendix S11. Canopy cover change matrix between 2008 and 2018 (area in sq. km)

| 2018 | 2008 | | | | | |
| --- | --- | --- | --- | --- | --- | --- |
|  | Area | NC | OC | DC | VDC | Grand Total |
|  | NC | 903.3 | 368.6 | 1.6 | 0.0 | 1273.5 |
|  | OC | 724.3 | 5276.9 | 205.2 | 2.4 | 6208.8 |
|  | DC | 1.9 | 828.3 | 700.1 | 48.8 | 1579.1 |
|  | VDC | 0.0 | 6.5 | 199.3 | 584.0 | 789.8 |
|  | Grand Total | 1629.5 | 6480.3 | 1106.2 | 635.2 | 9851.2 |
| Note: No Canopy (NC); Open Canopy (OC), Dense Canopy (DC), Very Dense Canopy (VDC) | | | | | | |

Appendix S12. Percentage covers of protected area (PA) in different potential habitats of gibbon in the study area.

| **Model threshold** | **Total Area (km^2^)** | **Area of PAs (km^2^)** | **% PA cover** |
| --- | --- | --- | --- |
| **High Potential** | 6009.1 | 1065.27 | 17.73 |
| **Moderate Potential** | 1949.3 | 536.27 | 27.51 |
| **Low Potential** | 1892.7 | 449.29 | 23.74 |
| **Total** | 9851.2 | 2050.83 | 20.82 |

Appendix S13. Canopy area statistics in protected and non protected zones of the study area and its respective percentage out of different canopy category

| **Year** | **Protected** | | | | **Non protected** | | | |
| --- | --- | --- | --- | --- | --- | --- | --- | --- |
|  | **No Canopy** | **Open Canopy** | **Dense Canopy** | **Very Dense Canopy** | **No Canopy** | **Open Canopy** | **Dense Canopy** | **Very Dense Canopy** |
| **Area (km^2^)** | | | | | | | | |
| **2008** | 112.19 | 715.47 | 470.36 | 617.38 | 1517.31 | 5764.83 | 635.84 | 17.82 |
| **2018** | 220.42 | 549.41 | 395.01 | 750.55 | 1053.08 | 5659.39 | 1184.09 | 39.25 |
| **2008-2018** | -108.23 | 166.06 | 75.35 | -133.17 | 464.23 | 105.44 | -548.25 | -21.43 |
| **Percentage canopy out of total category area** | | | | | | | | |
| **2008** | 6.88 | 11.04 | 42.52 | 97.19 | 93.12 | 88.96 | 57.48 | 2.81 |
| **2018** | 17.31 | 8.85 | 25.01 | 95.03 | 82.69 | 91.15 | 74.99 | 4.97 |
| **Percentage canopy out of the total study area** | | | | | | | | |
| **2008** | 1.14 | 7.26 | 4.77 | 6.27 | 15.40 | 58.52 | 6.45 | 0.18 |
| **2018** | 2.24 | 5.58 | 4.01 | 7.62 | 10.69 | 57.45 | 12.02 | 0.40 |

Appendix S14. Fragmentation statistics of the identified forest fragments in the high potential gibbon habitat in the Upper Brahmaputra landscape. Patches are identified from Dense and Very Dense Canopy forest map of 2008 and 2018.

| **Class metrics** | **2008** | | **2018** | |
| --- | --- | --- | --- | --- |
|  | **Dense Canopy** | **Very Dense Canopy** | **Dense Canopy** | **Very Dense Canopy** |
| Class Area (km^2^) | 777.81 | 553.69 | 1221.88 | 69287.50 |
| Percent of Landscape (%) | 58.42 | 41.58 | 63.81 | 36.19 |
| Number of patches | 658 | 69 | 840 | 71 |
| Patch Density | 0.49 | 0.05 | 0.44 | 0.04 |
| Largest Patch Index (%) | 5.22 | 11.35 | 18.42 | 9.46 |
| Total Edge (km) | 776.25 | 776.25 | 729.50 | 729.50 |
| Edge Density (m/ha) | 5.83 | 5.83 | 3.81 | 3.81 |
| Landscape Shape Index | 31.77 | 9.83 | 35.84 | 8.59 |
| Mean Patch Size (km^2^) | 1.18 | 8.02 | 1.45 | 9.76 |
| Mean Contiguity Index | 0.22 | 0.36 | 0.23 | 0.34 |
| Mean Euclidian Nearest Neighbour Distance (m) | 864.83 | 1242.10 | 818.99 | 1646.21 |
